# Benchmarking Ligand-Based Virtual High-Throughput Screening with the PubChem Database

**DOI:** 10.3390/molecules18010735

**Published:** 2013-01-08

**Authors:** Mariusz Butkiewicz, Edward W. Lowe, Ralf Mueller, Jeffrey L. Mendenhall, Pedro L. Teixeira, C. David Weaver, Jens Meiler

**Affiliations:** Department of Chemistry, Pharmacology, and Biomedical Informatics, Center for Structural Biology, Institute of Chemical Biology, Vanderbilt University, Nashville, TN 37232, USA

**Keywords:** virtual screening, machine learning, quantitative structure-activity relations (QSAR), high-throughput screening (HTS), cheminformatics, PubChem, BCL

## Abstract

With the rapidly increasing availability of High-Throughput Screening (HTS) data in the public domain, such as the PubChem database, methods for ligand-based computer-aided drug discovery (LB-CADD) have the potential to accelerate and reduce the cost of probe development and drug discovery efforts in academia. We assemble nine data sets from realistic HTS campaigns representing major families of drug target proteins for benchmarking LB-CADD methods. Each data set is public domain through PubChem and carefully collated through confirmation screens validating active compounds. These data sets provide the foundation for benchmarking a new cheminformatics framework BCL::ChemInfo, which is freely available for non-commercial use. Quantitative structure activity relationship (QSAR) models are built using Artificial Neural Networks (ANNs), Support Vector Machines (SVMs), Decision Trees (DTs), and Kohonen networks (KNs). Problem-specific descriptor optimization protocols are assessed including Sequential Feature Forward Selection (SFFS) and various information content measures. Measures of predictive power and confidence are evaluated through cross-validation, and a consensus prediction scheme is tested that combines orthogonal machine learning algorithms into a single predictor. Enrichments ranging from 15 to 101 for a TPR cutoff of 25% are observed.

## 1. Introduction

The development of quantitative structure activity relationship (QSAR) models in ligand-based computer-aided drug discovery (LB-CADD) has shown practical value for *in silico* (virtual) high-throughput screening (HTS) to identify potential hit compounds, *i.e*., compounds that share a biological activity of interest [1]. However, the predictive power of QSAR models depends not only on molecular descriptors of chemical structure and mathematical models, but foremost on the size, quality, and composition of the training data. An increased need for QSAR analysis emerged with the advent of HTS in academic research [2]. Cost increases linearly with the number of compounds tested in the HTS experiment and the number of compounds physically available at one site is limited [3]. LB-CADD has the potential to reduce these costs in a resource-limited academic environment. 

Public databases such as PubChem [4] contain biological activities for several hundred thousands of compounds tested against different biological targets [5]. Nevertheless, the number of compounds tested in a HTS experiment is typically at least two orders of magnitude smaller than the tens of millions of drug-like small molecules listed in PubChem. The space of possible drug-like molecules is even larger and estimated to be 10^30^–10^60^ [6]. LB-CADD has the potential to increase and diversify the chemical space tested.

From a methods perspective, increased public availability of large HTS data sets enables thorough benchmarking of existing LB-CADD methods and triggers the development of new cheminformatics tools; it will ultimately contribute to the fundamental understanding of protein-small molecule recognition and enhance the use of small molecule tools in biology. LB-CADD is particularly attractive in the resource-limited environment of academia as it reduces the cost and increases quality of drug discovery and/or probe development for rare or neglected diseases.

### 1.1. Quantitative Structure Activity Relationships Relate Chemical Structure and Biological Activity

QSAR models seek to correlate the often complex non-linear relationship of chemical structure with the biological activity for a protein target [7,8]. Dudek *et al*. [9] and Du *et al*. [10] provide an in-depth overview of current QSAR methods. Hansch *et al*. pioneered classical QSAR by investigating the biological activity of a set of compounds in relation to their corresponding physicochemical properties (hydrophobic, electronic, and steric effects) using linear regression models [11,12]. Modern QSAR techniques employ fingerprints and 2D/3D descriptors coupled with machine learning (ML) methods [13,14].

### 1.2. Molecular Descriptors Numerically Encode Chemical Structure

The descriptors employed in this study (scalar, 2D/3D auto-correlation, radial distribution functions) are fragment-independent and transformation invariant. Fragment-independent molecular descriptors can encode the chemical structure of small molecules in a vector of constant length independent of compound size, composition, or position in space. Radial distribution functions [15] were successfully employed to study the A_2A_ adenosine receptor agonist effect of 29 adenosine analogues [16]. A separate study focused on prediction of native receptor affinities of 38 vitamin D analogues [17] outperforming fragment-based molecular descriptors. Autocorrelation descriptors [18] were used to train ML models that predict CDK4/D inhibitory activity [19] and negative ionotropic activity of calcium entry blockers, among other applications [20]. 

### 1.3. Machine Learning Techniques Have Viable Impact on the Generation of QSAR Models

ML algorithms have shown exciting potential for developing QSAR models to predict biological activity data [21,22,23]. ML methods recognize complex patterns and derive a model based on training data acquired from experimentally screened compound libraries. Subsequently, large compound libraries can be screened virtually or *in silico* and ranked by predicted biological activity. This prioritizes a subset of compounds that is enriched for active molecules for acquisition or synthesis. 

Mueller *et al*. [24] applied artificial neural networks (ANNs) to identify novel positive allosteric modulators for mGlu5, a G-protein coupled receptor (GPCR) involved in neurological disorders like schizophrenia in two separate experiments. QSAR models were trained based on a high throughput screen of approximately 144,000 compounds. These models were used to virtually screen commercially available compound libraries to prioritize the most potent compounds. The top ranked compounds were experimentally validated resulting in a significant hit rate increase of 28.2% compared to an initial experimental hit rate of 0.94%. 

Golla *et al*. [25] successfully applied genetic and evolutionary algorithms to virtually screen for novel chemical penetration enhancers (CPEs) utilized through transdermal drug delivery. A set of 272 CPEs served as a pool for initial structures and QSAR model generation. A total of 4,834 molecules were generated by the genetic algorithm and 893 molecules were accepted having a score below a set threshold of 8. The study identified 18 novel CPEs that were experimentally evaluated for cytotoxicity and permeability, four of which express marginal to no toxicological effects.

In another study, Sun *et al*. [26] applied support vector machines in conjunction with 2D molecular descriptors to identify compounds involved in drug-induced phospholipidosis (PLD). PLD is implicated in intracellular accumulation of phospholipids and formation of concentric lamellar bodies. A set of 4,161 unique drug-like compounds from various small molecule libraries were evaluated in a quantitative HTS experiment. The resulting data was employed to train QSAR models. Using one third of the data as a training set, the final model achieved a prediction accuracy of 90% on the remaining two thirds of compounds. 

### 1.4. Consensus of QSAR Models Has Potential to Improve Prediction Accuracy

Combination of different ML models can reduce the prediction error by compensating for the misclassification of any single predictor with the consensus of the remaining models [27]. Simmons *et al*. [28] compared several ML and chemometric methodologies used to develop ensemble classifiers on data sets derived from *in vivo* HTS campaigns. Model performance was compared using false negative and false positive error profiles. Ensemble classifiers constructed from methods like ANNs or DTs achieved true positive rates of over 80% in the top 1.4% of the ranked list with false positive rates between 5%–7%.

Svetnik *et al*. [29] introduced a procedure for building a sequence of predictive models using a Random Forest approach [30]. Each model is fitted to the gradient of a loss function in a stage-wise manner to analyze ten cheminformatics data sets. Results are comparable to those of other ensemble learning methods such as Bagging and Boosting and outperform regular decision trees with accuracy rates of over 80%.

On the other hand, Hewitt *et al*. [31] constructed QSAR consensus models based on Genetic Algorithms on smaller data sets for silastic membrane flux, toxicity of phenols to Tetrahymena pyriformis, acute toxicity to the fathead minnow and flash point. The data set sizes ranged from 250 to 605 compounds. The results suggest only marginal benefit for consensus models compared to a single model predictor.

### 1.5. Significance

The objective of this manuscript is three-fold: (1) To compose a comprehensive benchmark set for ligand-based computer-aided drug discovery (LB-CADD)—*i.e*., cheminformatics. While PubChem is available since 2004, only now it grew to the size and quality needed to assemble a benchmark set of realistic HTS experiments, where actives have been confirmed experimentally, a wide range of relevant drug targets is spanned, and all data is available in the public domain. The data sets are carefully post-processed and made available so as to establish a benchmark for developing LB-CADD methods at www.meilerlab.org/qsar_pubchem_benchmark_2012. (2) To substantiate anecdotal and isolated findings on best practices in LB-CADD. Cheminformatics studies have been published comparing different ML methods, testing different approaches to descriptor selection, and using consensus modeling approaches (see above). However, conclusions derived from these studies were often limited by small and/or unrealistic toy data sets or by studying only one of the aforementioned aspects in isolation. In result correlations between parameters remain uncertain, over-training and narrow application range on one class of target proteins are a major concern. The present study overcomes these limitations. (3) To introduce a cheminformatics framework BCL::ChemInfo that is freely available for non-commercial use. It exposes a variety of methods for molecular descriptor selection, and ML techniques including Artificial Neural Networks (ANN) [32,33,34], Support Vector Machines (SVM) with an extension for regression [35,36,37,38], Decision Trees (DT) [39,40,41], and Kohonen networks (KN) [42,43,44]. It also enables consensus predictions from different ML models.

## 2. Results and Discussion

### 2.1. Compilation of Validated PubChem HTS Screens Provides Benchmark Data Sets for Training QSAR Models

PubChem provides publically available libraries of small organic molecules that have been tested in a diverse set of HTS experiments. Primary screens often include many false-positive hit compounds which display a response in initial assay experiments but are inactive in confirmatory experiments. We focus on HTS experiments with a single well-defined biological target protein. With respect to the desired target, these hit compounds may include non-binders that act on a different component of the assay or binders that are non-specific to the target and recognize other biological molecules. To minimize the number of false-positive hit compounds we compiled nine data sets applying the following criteria: the HTS experiment must target one specific protein and contain a minimum of 150 *confirmed* active compounds. Further, we chose a diverse set of PubChem assays focused on pharmaceutically relevant small molecule protein targets such as GPCRs, ion channels, transporters, kinase inhibitors, and enzymes. All PubChem assays are identified by PubChem summary id (SAID) of the primary protein target and describe a collection of confirmatory screens for active compounds given by PubChem assay ids (AID). It proved critical to go through a detailed manual verification of the HTS experiments performed and collate PubChem raw data to arrive at high-quality data sets. Complete data sets and their compilation protocols are provided in the Experimental Section (Section 3.1). We propose that the data sets presented here can serve as a benchmark for further cheminformatics method development. An overview with statistics of all PubChem data sets can be found in Table 1. The data sets are made available at www.meilerlab.org/qsar_pubchem_benchmark_2012. 

**Table 1:** Overview of PubChem biological assays and data set statistics.

Protein Target Class	Protein Target	PubChem Summary Assay ID SAID	Number Actives		NumberInactives	Hit Rate	Inactives -to-ActivesRatio
**GPCR**							
	Orexin1 Receptor	435008		230	218,071	0.11%	948
	M1 Muscarinic Receptor	1798		188	61,661	0.30%	327
	M1 Muscarinic Receptor	435034		448	61,407	0.73%	138
**Ion Channel**							
	Potassium Ion Channel Kir2.1	1843		172	301,473	0.06%	1,752
	KCNQ2 potassium channel	2258		213	302,351	0.07%	1,419
	Cav3 T-type Calcium Channels	463087		703	100,210	0.70%	143
**Transporter**							
	Choline Transporter	488997		252	302,246	0.08%	1,199
**Kinase Inhibitor**							
	Serine/Threonine Kinase 33	2689		172	319,821	0.05%	1,859
**Enzyme**							
	Tyrosyl-DNA Phosphodiesterase	485290		292	344,477	0.08%	1,179

### 2.2. Machine Learning Algorithms Relate Chemical Structure to Biological Activity

Three supervised (ANN, SVM, DT) and one unsupervised (KN) ML approaches were evaluated to predict biological activity on confirmatory HTS assay data. ML methods can describe non-linear relations and recognize patterns within large sets of numerical descriptors. Trained models can adapt to complex interrelations and are capable of detecting even small signals at high noise levels. Likewise, non-linear methods are applied when no simple mathematical model can be assumed, many influencing factors interact, and the experimental uncertainty is high.

### 2.3. Quality Measures Assess the Predictive Power of Machine Learning Algorithms

The low ratio of active:inactive compounds in HTS data sets (typically 1:100–1:1000) leaves unbiased quality measures (e.g., classification accuracy) inappropriate for comparing results across different data sets. To facilitate meaningful comparisons of results across data sets, the integral of true-negative-rate (specificity, y-axis) and true-positive-rate (sensitivity, x-axis) (TNR-TPR) was chosen as an objective function for QSAR model training. It represents a 90° clock-wise rotation of the traditional receiver operating characteristic (ROC) curve [45]. The integral is identical to the well-known area under the curve (AUC) value. The TNR-TPR plot shows the accuracies of a model at predicting actives and inactives on separate axes, and is thus independent of the active:inactive ratio. It thereby preserves the key advantage of the ROC curve. However, it also eliminates a key disadvantage of the ROC curve: after virtual screening only a small fraction of compounds—the ones with predicted high activity—will be considered. How many actives are among these compounds depends on the very initial slope of the ROC curve which is difficult to measure with an integral as the optimal cutoff value on the x-axis tend to be very small (for example 10^−3^) and data set dependent, *i.e*., it needs to be adjusted for optimal performance. In the past the x-axis has therefore been often plotted on a logarithmic scale. For TNR-TPR plots the integration is performed from 0 to the desired TPR value—*i.e*., the fraction of actives recognized as such, for example 25% or 50%. The integral instead of the slope is now the determinant of model quality. In contrast the slope analyzed for ROC curves at a single point the integral computed for TNR-TPR curves presents a more robust measure for model quality (see Experimental Section). To facilitate comparison we report enrichment (ENR) as additional quality measure—*i.e*., what is the ratio of active compounds after virtual screening compared to the initial HTS experiment. Enrichment correlates with the slope of the ROC curve. 

### 2.4. QSAR Model Quality Depends Critically on the Selection of Optimal Descriptor Set

To systematically add the most significant descriptor elements for signal to noise increase, three descriptor selection methods were employed: Information Gain (IG), F-Score (FS), and Sequential Forward Feature Selection (SFFS). A total of 60 numerical descriptor groups were available for this study with a total of 1,284 descriptor values (see supplementary materials Table S1). All three methods were used separately for each ML technique and PubChem data set. 

To cope with the computational expense associated with each descriptor selection technique, reduced data sets were created for the training and monitoring data sets containing always all available active compounds. Sets of 30,000 and 10,000 inactive compounds for the training and monitoring partitions, respectively, were chosen randomly from each HTS data set. The independent data set was not altered. The optimal descriptor set is defined by the largest average integral of the TNR-TPR curve over all cross-validation experiments. The descriptor selection process aims to identify the combination of properties and encoding functions that describe the structural features of the pharmacophore best and hence yield the best QSAR model. The selection process allows for models with fewer degrees of freedom and therefore reduces training time, limits number of data points needed for training, and improves signal to noise. For every descriptor selection method, ML algorithm, and PubChem data set a comparison of the integral beneath the TNR-TPR curve was evaluated by assessing 10 × 9-fold cross-validated models using the optimal descriptor set shown in Table 2.

**Table 2:** Descriptor selection results for Information Gain (IG), F-Score (FS), and Sequential Feature Forward Selection (SFFS) applied to each ML technique paired with each PubChem HTS data set. Results for the integral of the TNR-TPR curve are presented. The mean and standard deviation for each SAID (row) and each descriptor selection approach (column) is given.

	ANN			SVM			DT			KN			
PubChem	IG	FS	SF	IG	FS	SF	IG	FS	SF	IG	FS	SF	Mean
SAID			FS			FS			FS			FS	(Stdev)
435008	0.79	0.81	0.80	0.84	0.84	0.85	0.77	0.77	0.77	0.77	0.77	0.77	0.80 (0.03)
1798	0.68	0.68	0.64	0.74	0.73	0.76	0.72	0.68	0.52	0.70	0.72	0.68	0.69 (0.06)
435034	0.79	0.80	0.80	0.83	0.82	0.85	0.74	0.74	0.50	0.75	0.76	0.78	0.76 (0.09)
2258	0.80	0.80	0.83	0.84	0.84	0.85	0.78	0.75	0.77	0.75	0.76	0.79	0.80 (0.04)
1843	0.91	0.90	0.92	0.92	0.91	0.92	0.86	0.84	0.83	0.86	0.83	0.86	0.88 (0.04)
463087	0.84	0.86	0.86	0.88	0.89	0.89	0.82	0.81	0.82	0.75	0.77	0.81	0.83 (0.05)
488997	0.76	0.75	0.75	0.79	0.81	0.82	0.77	0.74	0.75	0.74	0.75	0.76	0.77 (0.03)
2689	0.92	0.92	0.92	0.92	0.93	0.93	0.88	0.86	0.86	0.88	0.86	0.85	0.89 (0.03)
485290	0.83	0.84	0.85	0.86	0.86	0.86	0.82	0.84	0.76	0.80	0.80	0.75	0.82 (0.04)
Mean (Stdev)	0.81 (0.07)	0.82 (0.07)	0.82 (0.09)	0.85 (0.06)	0.85 (0.06)	0.86 (0.05)	0.79 (0.05)	0.78 (0.06)	0.73 (0.13)	0.78 (0.06)	0.78 (0.04	0.78 (0.05)	

The mean value of the TNR-TPR integral comparing the performance of ML algorithms across different descriptor selection methods ranged from 0.73–0.79 (DT), 0.78 (KN), to 0.81–0.82 (ANN) and 0.85–0.86 (SVM). These results provide a clear distinction of prediction performance between individual single predictors. The mean performance of a cross-validated QSAR model considering each PubChem data set individually ranged from 0.69 (SAID 1798) to 0.89 (SAID 2698); standard deviations ranged from 0.03 to 0.09. ANN and SVM typically outperform DT and KN with mean integral values above the baseline independent of the chosen data set.

### 2.5. Consensus Prediction of Machine Learning Techniques Increases Prediction Accuracy

A consensus prediction was obtained by averaging the output of several ML techniques (see Experimental). For every data set, all possible combinations of ML methods were assessed using the previously determined optimal descriptor set for each ML method (see Table 3). We ranked QSAR model performance using the achieved ENR value. The best single predictor is listed at the end of each ranking if not present among the top three consensus predictors. 

Consensus models consistently outperform QSAR models that rely on a single ML method. Misclassifications of single predictors are generally extenuated and therefore consensus predictors achieve increased prediction accuracy. Normalizing the ENR increase between the best consensus predictor and best single predictor by the inactives-to-actives ratio reveals differences ranging from −0.21% (SAID 438005, IG) to 4.75% (SAID2689, SFFS). In all but a single case the consensus predictor outcompetes individual predictors suggesting that consensus prediction provides a benefit although it is small. The overall ENR increase of consensus predictors compared to the theoretical maximum ENR appears to be marginal, though significant when compared to the hit rate of the HTS experiment (see Table 1). Conflicting findings from previous studies [27,28,29,30,31] can be attributed to a limited benchmark. 

**Table 3:** The top two ranking (#r) consensus QSAR models are presented for each PubChem data set (SAID) and descriptor selection techniques: F-Score (FS), Information gain (IG), and sequential feature forward selection (SFFS). Every model is evaluated by the integral of the TNR-TPR curve with a TPR rate of 0.00 to 0.25 (INT). The ranking is ordered by Enrichment (ENR). The best single predictor is shown for comparison. The ENR difference of the best consensus predictor compared to the best single predictor normalized by the inactives-to-actives ratio is given (Diff).

SAID	FS	#r	INT	ENR	IG	#r	INT	ENR	SFFS	#r	INT	ENR
435008	ANN DT KN SVM	1	0.249	27	SVM	1	0.245	24	DT SVM	1	0.247	40
	SVM	2	0.245	26	DT SVM	2	0.245	22	SVM	2	0.246	39
	DT SVM	3	0.245	26	KN SVM	3	0.245	22	ANN DT SVM	3	0.249	27
	Diff	-	-	0.11%	Diff	-	-	0.21%	Diff	-	-	0.11%
1798	ANN DT KN SVM	1	0.240	10	ANN DT KN SVM	1	0.241	15	ANN DT KN SVM	1	0.243	7
	ANN DT SVM	2	0.240	9	ANN KN SVM	2	0.241	10	ANN KN SVM	2	0.243	7
	SVM	6	0.240	8	SVM	8	0.242	7	SVM	8	0.244	5
	Diff	-	-	0.61%	Diff	-	-	2.45%	Diff	-	-	0.61%
435034	ANN SVM	1	0.246	18	ANN SVM	1	0.245	17	ANN SVM	1	0.246	18
	ANN DT SVM	2	0.246	17	ANN DT SVM	2	0.245	16	ANN DT SVM	2	0.246	18
	SVM	7	0.245	14	SVM	4	0.246	16	SVM	3	0.246	17
	Diff	-	-	2.90%	Diff	-	-	0.72%	Diff	-	-	0.72%
1843	ANN DT KN	1	0.249	54	ANN DT SVM	1	0.250	68	ANN DT KN SVM	1	0.250	50
	DT KN SVM	2	0.249	45	ANN DT KN SVM	2	0.250	67	ANN DT SVM	2	0.250	45
	SVM	11	0.249	32	SVM	10	0.250	45	ANN	11	0.250	30
	Diff	-	-	1.26%	Diff	-	-	1.31%	Diff	-	-	1.14%
2258	ANN DT	1	0.241	28	ANN DT	1	0.241	34	ANN DT KN	1	0.244	65
	ANN DT SVM	2	0.246	26	ANN DT KN SVM	2	0.246	33	DT KN SVM	2	0.249	49
	SVM	8	0.246	18	DT	6	0.238	23	SVM	10	0.249	28
	Diff	-	-	0.70%	Diff	-	-	0.78%		-	-	2.61%
463087	ANN SVM	1	0.250	23	ANN DT KN SVM	1	0.250	19	ANN KN SVM	1	0.250	29
	SVM	2	0.250	22	ANN DT SVM	2	0.250	18	ANN DT KN SVM	2	0.250	28
	DT SVM	4	0.250	21	SVM	8	0.250	17	SVM	8	0.250	24
	Diff	-	-	0.70%	Diff	-	-	1.40%	Diff	-	-	3.50%
2689	ANN DT KN SVM	1	0.248	74	ANN DT KN SVM	1	0.248	58	ANN DT SVM	1	0.249	101
	ANN DT SVM	2	0.248	63	ANN DT SVM	2	0.248	54	ANN DT KN SVM	2	0.249	91
	ANN	10	0.250	42	SVM	10	0.248	41	ANN	10	0.248	44
	Diff	-	-	2.67%	Diff	-	-	1.42%	Diff	-	-	4.75%
488997	ANN DT SVM	1	0.246	20	DT KN SVM	1	0.244	14	ANN DT KN SVM	1	0.243	49
	ANN DT KN SVM	2	0.247	19	ANN DT KN	2	0.241	13	ANN KN SVM	2	0.243	44
	SVM	6	0.245	15	DT	7	0.242	12	SVM	11	0.244	31
	Diff	-	-	0.27%	Diff	-	-	0.11%	Diff	-	-	0.97%
485290	ANN DT KN	1	0.241	64	DT KN SVM	1	0.245	71	ANN SVM	1	0.244	30
	DT KN SVM	2	0.245	58	ANN DT KN SVM	2	0.246	60	ANN DT SVM	2	0.244	28
	SVM	11	0.244	38	SVM	12	0.245	36	SVM	4	0.244	26
	Diff	-	-	2.22%	Diff	-	-	2.96%	Diff	-	-	0.28%

## 3. Experimental

### 3.1. Determination of Confirmatory High-Throughput Screening Data Sets for Diverse Protein Targets

Publicly available libraries of small organic molecules from a diverse set of HTS experiments were obtained from PubChem. The following listing of PubChem assays identifies the PubChem summary id (SAID) of the primary protein target and describes the determination of active compounds from confirmatory screens given by PubChem assay ids (AID). The inactive compounds are taken from the corresponding primary assay.

#### 3.1.1. GPCR: Antagonist of the Orexin 1 Receptor (SAID 435008)

The GPCR Orexin 1 plays a role in behavioral plasticity, the sleep-wake cycle, and gastric acid secretion [46,47]. Three primary screens, AID 485270, AID 463079, AID 434989, were conducted to identify antagonists of Orexin 1 receptor. AID 485270 is a FRET-based cell-based assay [48] identifying compounds that inhibit Orexin 1 receptor activity. AID 463079 is a cell-based assay using a parental CHO cell line identifying compounds that non-selectively inhibit G_q_ signaling. Here, compounds are tested for inhibition of G_q_ activity using the parental CHO cell line without transfection of the GPCR. AID 434989 is a fluorescence-based cell-based assay identifying compounds with inhibitory activity of the Orexin 1 receptor. These compounds are dispensed onto CHO cells with transfected human Orexin 1 receptors to gauge calcium mobilization by a fluorescent indicator dye. Inhibitors revealed by the primary screen AID 485270 were confirmed by the counter screen AID 492964 through a Homogeneous Time Resolved Fluorescence (HTRF)-based cell-based assay. Further, resulting inhibitors from assay AID 492964 were investigated by the counter screen AID 493232 that tested for non-selectivity due to inhibition of G_q_ activity. It applied a HTRF-based cell-based assay to identify antagonists of the parental CHO-K1 cell line. Subsequently, the validation assay AID504701 identified compounds being active in primary screen AID 483270, confirmed in assay AID 493964, but inactive in counter screen AID 493232 to exclude compounds with non-selectivity due to inhibition of G_q_ activity. AID504701 applied an HTRF-based cell-based dose response assay to identify antagonists of the Orexin 1 receptor. Another validation screen, AID 492965, confirmed compounds, active in AID 434989 and inactive in primary screen AID 463079, being non-selective inhibitors of G_q_ signaling. The applied assay was a fluorescence-based cell-based HTS confirmation assay. A second primary assay, AID 434989, screened for agonists of the Orexin 1 receptor with validation of inhibitory activity by AID 492963. AID 492963 used a fluorescence-based cell-based HTS confirmation assay to identify antagonists of the Orexin 1 receptor. A more specific assay, AID504699, identified compounds that are active in AID 434989 and AID 492963 but being inactive against the parental cell line tested in a third primary screen AID 463079. AID504699 applied a fluorescence-based cell-based HTS confirmation assay to identify antagonists of the Orexin 1 receptor. 

Combining the active compounds of the most refined assays, AID504701 and AID504699, resulted in a total of 234 active compounds excluding an overlap of 155 molecules.

#### 3.1.2. GPCR: Allosteric Modulators of M1 Muscarinic Receptor: Agonist (SAID 1798)

The G_q_-coupled GPCR M1 Muscarinic Receptor [49,50,51,52] is a seven-transmembrane domain receptors whose modulation has significant impact in treatment of cognitive degeneration associated with Alzheimer’s Disease and schizophrenia. The same set of compounds was screened for positive (AID626) and negative (AID628) allosteric modulation of the M1 Muscarinic receptor. Agonistic modulators of M1 Muscarinic receptor were confirmed by screen AID 1488 applying a cell-based fluorometric calcium assay. A second counter screen AID 1741, using the same assay settings as AID 1488, evaluated these compounds for cross-activity with M4 muscarinic receptor. The final set of selective positive allosteric modulators of M1 was obtained by removing compounds active in AID 1741 from the compounds active in AID 1488 resulting in 188 compounds.

#### 3.1.3. GPCR: Allosteric Modulators of M1 Muscarinic Receptor: Antagonist (SAID 435034)

Negative modulators of M1 muscarinic receptors (AID628) [53,54] were confirmed by screen AID677 through a cell-based fluorometric calcium assay. AID859 confirmed activity on rat M1 receptor. The counter screen AID860 removed non-selective compounds being active also at the rat M4 receptor. AID859 and AID860 employed the same assay type as AID677 and AID628. To remove the non-selective actives having a different target then the rat and human M1 receptor, the final set of active compounds was obtained by subtracting active compounds of AID860 from those in AID677, resulting in 448 total active compounds.

#### 3.1.4. Ion Channel: Potentiators of KCNQ2 Potassium Channel (SAID 2258)

Voltage-gated potassium channels, like KCNQ2 [55,56], have important neuronal functionality in excitement and resting states of cells. This target institutes a new avenue for drugs attempting to treat cancer, autoimmune diseases, metabolic, neurological, and cardiovascular disorders. The primary screen AID 2239 identified potentiators of KCNQ2 potassium channel through measurements of intracellular thallium, gauged by the intensity of a thallium-sensitive fluorescent dye. A confirmatory screen AID 2287 validated active compounds to be potentiators. Counter screens identified false positive compounds showing response for CHO-K1 cell activity (AID 2282), non-specific effects on KCNQ1 (AID 2283) and response in KCNQ2-W236L-CHO cells (AID 2558). All confirmatory and counter screens applied the same experimental conditions as in the primary screen.

The final set of 213 active compounds was acquired by removing the active compounds of AID 2282, AID 2283 and AID 2558 from the confirmatory screen active set of compounds (AID 2287). 

#### 3.1.5. Ion Channel: Identification of Compounds that Inhibit Inward-Rectifying Potassium Ion Channel K_ir_2.1 (SAID 1843)

The K_ir_2.1 inward-rectifier potassium ion channel is recognized as a target in the treatment of cardiovascular, neurological, renal and metabolic disorders [57,58,59]. The primary assay AID 1672 identified inhibitors for the inward-rectifying potassium ion channel Kir2.1. The assay uses a HEK293 cell line with stably expressed Kir2.1 channels where test compounds are gauged by intracellular thallium through thallium-sensitive fluorescent dye. The validation screens AID 2032 and AID 463252, both confirmed active compounds from the primary screen showing inhibition of K_ir_2.1. While AID 2032 used the same assay experiment as the primary screen, AID 463252 applied an automated electrophysiology assay for Kir2.1. The counter screens AID 2105, AID 2345, AID 2236, and AID 2329 identified active compounds exhibiting non-specific binding effects against K_ir_2.1. AID 2105 is a counter screen to the primary screen and evaluated active compounds against their non-specific effects on parental HEK293 cells of Kir2.1-HEK293 cells. AID 2236 tested compounds identified in the primary screen assay for effects on hERG CHO cells. AID 2345 assess compounds identified as active in an independent primary screen assay (PubChem AID 2239) for non-specific effects on the Kir2.1 stably expressed HEK293 cells as well as on KCNQ2 potassium channels.

The final set of 172 active compounds was assembled by subtracting the actives in AID 2105, AID 2345, AID 2236, and AID 2329 from the molecules found active in both, AID 2032 and AID 463252.

#### 3.1.6. Ion Channel: Inhibitors of the of Cav3 T-type Calcium Channels (SAID 463087)

The transient-type (T-type) calcium channel containing one of three α1 subunits (Cav3) is part of the voltage-gated potassium channel family and has suggested involvement in epileptics and pulmonary hypertension [60,61,62,63]. The primary screen AID 449739 identified inhibitors of Cav3 T-type calcium channels measuring calcium fluorescence change in a Cav3.2 expressing cell line. AID 489005 is a counter screen validating active compounds of the primary screen using the same assay. Four follow-up screens were performed to confirm inhibitory effects on smaller sets of compounds involving AID 493021, AID 493022, AID 493023, and AID 493041. All were confirmatory and tested dose-response through 11-point 3-fold experiments using the same assay conditions as the primary screen. 

The final set of 703 active compounds was acquired by subtracting the inactive compounds of the latter follow-up screens from the actives in the validation screen AID 498005. Taking just the actives of the four follow-up screens would have violated the established benchmark data set requirements.

#### 3.1.7. Transporter: Inhibitors of the Choline Transporter (CHT, SAID 488997)

Choline has many physiological functions throughout the body that are dependent on its available local supply [64,65]. Its transport is required for cellular membrane construction and is the rate-limiting step for acetylcholine production. CHT is suggested a drug target involved in Alzheimer's Disease. The primary screen AID 488975 identified inhibitors of CHT. The counter screen AID 493221 is a validation screen to confirm the active compounds that inhibit CHT. It uses a choline-induced membrane potential assay measuring choline coupled sodium flow through CHT. Further, two additional validation screens reaffirmed activity of these compounds with 5-point concentration response curve (CRC) (AID504840) and 10-point CRC (AID588401) experiments. The screen AID 493222 evaluated remaining active compounds for non-specific activity in parental HEK293 cells. Finally, the reconfirmation screen AID602208 tested a selected set of compounds for 3H choline uptake. 

The final set of 254 active compounds was determined by the overlap of active compounds in screens AID 493221, AID504840, and AID588401 subtracting any non-specific hits from AID 49322 and all inactive compounds in the re-confirmation screen AID602208.

#### 3.1.8. Kinase Inhibitor: Inhibitors of Serine/Threonine Kinase 33 (STK33, SAID 2689)

The serine/threonine kinase, STK33, has been identified and shown to be required for the survival and proliferation of mutant KRAS-dependent cells involved in cancer [66]. The primary screen AID 2661 identified inhibitors of STK33 through preincubation of purified STK33 Kinase with potential inhibitors and kinase activity is measured through luminescent signal strength.

The counter screen AID 2821 reaffirmed active compounds from AID 2661 using the same experimental conditions as in the primary screen. AID504583 tested a subset of compounds for STK33 selectivity by measuring Protein Kinase A inhibition. Taken the actives in AID 2821 subtracted by the actives from screen AID504583 resulted in the final set of 172 active compounds.

#### 3.1.9. Enzyme: Inhibitors of Tyrosyl-DNA Phosphodiesterase 1 (TDP1, SAID 485290)

The inhibition of Human tyrosyl-DNA phosphodiesterase 1 (TDP1) has the potential to enhance anticancer activity of DNA topoisomerase I inhibitors [66,67,68,69]. The primary screen AID 485290 identified inhibitors of TDP1. The counter screen AID 489007 was used as a confirmation of the previously identified actives. AID 489007 used the AlphaScreen detection method [69] measuring the intensity of an enzyme cleavage reaction. The final set of 292 actives contains all compounds labeled as active in the counter screen AID 489007.

### 3.2. Numerical Representation of Biological Data Distinguishes Active from Inactive Compounds

The half maximal inhibitory and effective concentrations, IC_50_ and EC_50_ values, of active compounds from the HTS data ranged from 0.1 μM to 25 μM. Biological activity was not reported for every active compound, thus all active compounds without an assigned IC_50_ or EC_50_ value were categorized as actives with a representative value of 1 μM chosen from the actives concentration range. All inactive compounds were set to a biological activity value of 1 mM. Models were trained on pIC_50_ = −log_10_ (IC_50_/1 M), which ranges from 3 (for inactive molecules) to 4.6 to 7 (for molecules with IC_50_ = 25 to 0.1 µM). The same method was applied for pEC_50_ values. This procedure ensures that compounds without determined IC_50_/EC_50_ can be used for training while at the same time information on differential activity is leveraged when available. In our hands this procedure is superior to a pure binary classification in active/inactive (data not shown).

### 3.3. Numerical Description of Molecules for QSAR Model Development

A total of 1,284 numerical descriptors in 60 categories were implemented in this study (see supplementary materials Table S1). The 60 categories contain scalar descriptors such as molecular weight, number of hydrogen bond donors, -acceptors, octanol/water partition coefficient, total charge, and topological polar surface area. Nine additional chemical properties were computed for every atom including atom identities, σ-, π-, and total charges, σ-, π-, and lone pair electronegativities, effective atom polarizabilities, and VC2003 atom charges [70]. Three encoding functions (2D auto-correlation, 3D auto-correlation, radial distribution function) are paired with each of the chemical properties to yield 27 fingerprints [16,19]. In addition, each fingerprint is computed a second time applying van der Waals surface area as a weight factor. 3D conformations for all molecules were calculated with CORINA [71].

### 3.4. Monitoring Data Set is Used for Early Termination of Training Process

The oversampled data set had 80% of the data points employed in the actual training process. The number of training iterations was limited through early termination to counter “overfitting” of the ML model to the training data. A monitoring data set consisting of 10% of the data points was used to optimize all training parameters of the ML methods and to invoke early termination. The final 10% of the data points are set aside as an independent data set. It is not employed in the training process, but was evaluate by the final model. There was no overlap of compounds between training, monitoring, and independent data sets. 

### 3.5. The Integral of the True-Negative-Rate–True-Positive-Rate Curve is a Viable Quality Measure for QSAR Models

Similarly to a traditional receiver operating characteristic (ROC) curve, QSAR models were evaluated by means of a true-negative-rate - true-positive-rate (TNR-TPR) curve. It resembles a clockwise rotated ROC curve plotting the rate of true negatives TNR = *TN/N* = 1 − *FR/N* = *TN*/(*FP* + *TN*) (or specificity) versus the rate of true positives TPR = *TP/P* = *TP/*(*FN*+*TP*), also known as sensitivity. The diagonal represents performance of a random predictor and has an integral (area under the curve, AUC) of 0.5. The QSAR model progressively improves as the integral increases. For virtual screening where only a small fraction of a screened compound library will be tested experimentally, performance at high true positive rates (or low false positive rates) is most critical (see below). 

### 3.6. Enrichment Measures Ratio of Fraction of Active Compounds Predicted Above Actives Rate

QSAR models are most frequently applied in virtual screening experiments: activities are predicted for a large compound library (e.g., ~10^5^). Compounds are ranked by predicted activities to select a small fraction of compounds for experimental testing (e.g., 1% or ~10^3^). In this scenario it is important that the 1,000 compounds predicted as most active are actually active while the ranking of the other 99,000 compounds is of a lesser concern. This property of the QSAR model is not well reflected in the global AUC value as it only depends on the initial integral of the TNR-TPR curve. It is better analyzed through computation of enrichment:

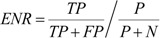
(1)


The value represents the factor by which the fraction of active compounds was increased through virtual screening above the background observed in the original HTS experiment.

### 3.7. Orthogonal Supervised and Unsupervised Machine Learning Algorithms Seek Optimal Biological Activity Predictions

A set of four supervised and unsupervised ML algorithms were implemented in this study. The first supervised algorithm is the Artificial Neural Networks (ANN). The utility of ANNs for classification is well-known in chemistry and biology [21,23,72,73,74,75]. Their architectural arrangement resembles the network structure of neurons. Layers of neurons are connected by weighted edges w_ji_. The input data x_i_ are summed according to their weights, an activation function is applied, and its output used as the input to neurons of the next layer. Simple propagation [32] was chosen as the weight update algorithm during the training process. The parameters *η* (learning rate) and α (momentum) were optimized prior to descriptor selection and again with the optimized descriptor set. 

Support Vector Machine (SVM) learning with extension for regression estimation [35,37] represents a supervised ML approach successfully applied in the past [21,76,77,78]. The core principles lay in linear functions defined in high-dimensional hyperspace [79], risk minimization according to Vapnik’s - intensive loss function, and structural risk minimization [38] of a risk function consisting of the empirical error and the regularized term. SVMs were trained using an initial penalty parameter C and kernel parameter γ of 1 and 0.1 respectively, during the descriptor optimization process. Upon identification of the optimal descriptor set, C and γ were optimized in a grid search approach for every data set. 

The decision tree (DT) learning algorithm [28,29,40] determines sets of rules to partition a given training data set. A partitioning algorithm gauges each successive split into subset or decision nodes with increased purity of one small molecule category. The splitting criterion is ascertained by the Gini coefficient [80]. The resulting model is a sequence of decisions involving single predictor variables that classify a given feature. The DT implementation in BCL::ChemInfo ranks outcomes based on the percent of actives that were mapped into that node during training.

The Kohonen network (KN) represents an unsupervised learning algorithm [42,43,44]. The KN clusters similar inputs into nodes on a spatial grid, thus forming a reduced-dimensional representation of the problem space. Each node represents a cluster of similar compounds based on a Gaussian neighbor kernel distance measure.

### 3.8. Cross-Validation Ascertains Robustness of QSAR Models

The active and inactive data sets are divided into ten equal-sized partitions. The first partition is specified as the independent data set which is constant during cross-validation. Of the remaining nine partitions a second partition is selected as the monitoring data set. The remaining eight subsets constitute the training data set. A different monitoring data set is chosen systematically for each iteration of the cross-validation. In a set of ten data partitions each of those ten partitions can be assigned as independent data set leaving nine possibilities of assigning one remaining data partition as the monitoring data set. This results in 10 × 9 = 90 possible model training configurations. All final models trained using the optimized descriptor sets in this study are 10 × 9 -fold cross-validated. This procedure still ensures that every molecule in the data set was part of an independent data partition at least once during cross-validation. Data sets for ANNs and SVMs were balanced by oversampling actives, while decision trees and KNs required no oversampling. 

To reduce the computational burden, all descriptor selection schemes use a 5 × 1 = 5 fold cross-validation set up, where the monitoring data partition is systematically incremented but only one independent data set configuration is evaluated. 

### 3.9. Selection of an Optimized Descriptor Set Guides QSAR Model Training

To reduce the total number of inputs to ML algorithms, it is advantageous to remove obsolete descriptors in order to minimize the number of degrees of freedom that need to be determined. Further, noise is reduced while the ratio of data points versus degrees of freedom increases. The determination of an optimal set of descriptors for each data set was evaluated by various selection methods such as Information gain [81], F-Score [82], and Sequential Forward Feature selection [83]. 

Information gain (IG) and F-Score (FS) score every descriptor column in the data set by statistical metrics that consider the actives/inactives composition. These scores can be compared: higher values indicate a higher discriminating power between active and inactive data points for a particular descriptor column. A particular advantage of these metrics over SFFS is that relatively few models need to be trained, because the scores are independent from model training.

IG measures the change of information entropy from the overall compound distribution of actives and inactives in one descriptor column compared to the entropy in each descriptor category itself. A higher information gain value of a descriptor column indicates higher discriminating information content.


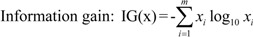
(2)

The variable *x_i_* represents *i* th feature of the combined active and inactive data sets. FS considers the mean and standard deviation of each descriptor column across active and inactive compounds

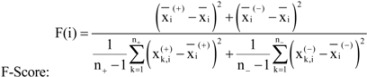
(3)
where 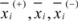 are the average of the *i* th feature of the whole, active, and inactive data sets, respectively; 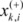 is the *i* th feature of the *k* th active instance, and 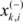 is the *i* th feature of the *k* th inactive instance. 

SFFS evaluates the objective function of trained models directly to arrive at an optimal descriptor set. This approach is a deterministic greedy search algorithm over all descriptor groups (see supplementary materials Table S1). Each round adds a single descriptor group to the descriptor set (initially, the empty set) selected in the previous round. Descriptor sets for the current round are then formed by adding each candidate descriptor group to the descriptor set selected in the previous round. Descriptors already present in the best descriptor group are ignored when creating the descriptor sets for a given round. Five-fold cross-validated models are trained followed by the evaluation of respective objective functions. The average objective function result is computed for each cross-validated model, and the descriptor set corresponding to the top performing models is selected as the best descriptor set for this round. This process is repeated until all features are selected or early terminated if no improved was determined for ten consecutive rounds. Finally, the best descriptor combination is chosen from the best performing model.

### 3.10. Consensus Predictions Seeks Improved Accuracies of Trained QSAR Models

The combination of different ML model predictions can reduce the overall prediction error by compensating for misclassification of a single predictor with the consensus of the remaining models [27]. Here, we evaluate the overall accuracy of all trained QSAR models by calculating average consensus of all predicted pIC_50_ or pEC_50_ values given in an independent data set:

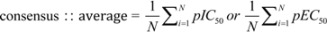
(4)

If the predicted pIC_50_ or pEC_50_ value is at or above a given cutoff the predicted activity of the molecule is evaluated as active. Consensus, prediction was performed over all cross-validated QSAR models, five cross-validation models for every of the four ML methods.

### 3.11. Implementation

All ML algorithms and all molecular descriptors were implemented in the BioChemistryLibrary (BCL). This software suite was developed in-house as an object-oriented library written in the programming language C++. It contains more than 1000 classes and approximately 500,000 lines of code. This library is the basis for BCL::ChemInfo and other modeling algorithms regarding small organic molecules and large molecular structures such as proteins. BCL::ChemInfo is a tailored method that streamlines data processing such as data set generation and cross-validation. The framework hosts a range of small molecule descriptors, descriptor selection strategies, and ML technologies. Speedups between 80 and 200 are achieved through OpenCL implementations of ANNs and SVMs used on graphics processing units (GPUs) hosted on an in-house CPU/GPU cluster. A command line interface is provided for easy access. Thus, no meta-language has to be learned like R or Matlab. BCL::ChemInfo is freely available for non-commercial use at www.meilerlab.org/bclcommons.

## 4. Conclusions

In this study, nine large data sets were assembled originating from realistic HTS experiments for a range of common drug target proteins including GPCRs, ion channels, transporters, kinase inhibitors, and enzymes. All data was drawn from the public domain through PubChem but carefully post-processed to include only confirmed active compounds. These data sets provide a foundation for developing and testing methods in LB-CADD. We further introduce a comprehensive framework for LB-CADD termed BCL::ChemInfo that is freely available for non-commercial use and exposes orthogonal ML approaches including ANNs, SVMs, DTs, and KNs. We confirm that the quality of QSAR models depends critically on selection of optimal molecular descriptors, composition of the training data, and the ML method itself. 

Further optimization was achieved by combining different ML methods into a consensus prediction to reduce false positives of each individual method. Theoretical enrichments ranging from 15 to 101 for a TPR cutoff of 25% are observed. The overall enrichment improvement normalized by maximal possible enrichment of consensus predictors compared to single predictors is up to 4.75%. We derive a ‘TNR-TPR curve’ from the common ROC analysis to better evaluate the quality of QSAR models at high TPR/FPR ratios.

LB-CADD or ‘cheminformatics’ is one strategy to reduce costs and increase size of the chemical space in resource-limited academic probe development efforts or drug discovery campaigns that target orphan or neglected diseases. This study shows that QSAR models prioritize compounds *in silico* thereby limiting the cost of HTS and hit-to-lead optimization. The availability of HTS data through PubChem allows for a comprehensive comparison of QSAR models, molecular descriptor selection, and training strategies. The data sets compiled in the present study are available for future cheminformatics method development at www.meilerlab.org/qsar_pubchem_benchmark_2012.

## Supplementary Materials

Supplementary materials can be accessed at: http://www.mdpi.com/1420-3049/18/1/735/s1.
