# Supplementary material for: Benchmarking Ligand-Based Virtual High-Throughput Screening with the PubChem Database
Source: Molecules. 2013 Jan 8;18(1):735–56. doi: 10.3390/molecules18010735 (PMC3759399; doi:10.3390/molecules18010735)
Supplement: Supplementary file 1 [file molecules-18-00735-s001.pdf]

Supplementary Materials

Benchmarking Ligand-Based Virtual High-Throughput Screening with the PubChem Database

Mariusz Butkiewicz, Edward W. Lowe Jr., Ralf Mueller, Jeffrey L. Mendenhall, Pedro L. Teixeira, C. David Weaver and Jens Meiler *

Department of Chemistry, Pharmacology, and Biomedical Informatics, Center for Structural Biology, Institute of Chemical Biology, Vanderbilt University, Nashville, TN 37232, USA

***** Author to whom correspondence should be addressed; E-Mail: jens.meiler@vanderbilt.edu; Tel.: +1-615-936-5662; Fax: +1-615-936-2211.

S1. Molecular Descriptors Numerically Encode Chemical Structure

A total of 60 descriptor groups with 1,284 numerical descriptors were implemented in this study (see Table S1). The 60 categories contain scalar descriptors such as molecular weight, number of hydrogen bond donors, -acceptors, octanol/water partition coefficient, total charge, and topological polar surface area. Nine additional chemical properties were computed for every atom including atom identities, σ-, π-, and total charges, σ-, π-, and lone pair electronegativities, effective atom polarizabilities, and VC2003 atom charges [1]. Three encoding functions (2D auto-correlation, 3D auto-correlation, radial distribution function) are paired with each of the chemical properties to yield 27 fingerprints [2,3]. In addition, each fingerprint is computed a second time applying van der Waals surface area as a weight factor.

Table S1. Overview of molecular descriptors by group number, category, name, and number of descriptor features.

#Group	Category	Name	#Features
	Scalar descriptors		
1		Molecular weight of compound	1
2		Number of hydrogen bonding acceptors derived from the sum of nitrogen and oxygen atoms in the molecule	1
3		Number of hydrogen bonding donors derived from the sum of N-H and O-H groups in the molecule	1
4		Topological polar surface area in Å^2 of the molecule derived from polar 2D fragments	1
5		Octanol/water partition coefficient in log units	1
6		Total charge of the molecule	1
	Vector descriptors		
7	2D autocorrelation	Atom identity	11
8		Sigma charge	11
9		Pi charge	11
10		Total charge	11
11		Sigma electronegativity	11
12		Pi electronegativity	11
13		Lone pair electronegativity	11

Table S1. *Cont.*

#Group	Category	Name	#Features
14		Polarizability	11
15		VC/2003 partial atom charges	11
16	2D autocorrelation weighted with van der Waals surface area	Atom identity	11
17		Sigma charge	11
18		Pi charge	11
19		Total charge	11
20		Sigma electronegativity	11
21		Pi electronegativity	11
22		Lone pair electronegativity	11
23		Polarizability	11
24		VC/2003 partial atom charges	11
25	3D autocorrelation	Atom identity	12
26		Sigma charge	12
27		Pi charge	12
28		Total charge	12
29		Sigma electronegativity	12
30		Pi electronegativity	12
31		Lone pair electronegativity	12
32		Polarizability	12
33		VC/2003 partial atom charges	12
34	3D autocorrelation weighted with van der Waals surface area	Atom identity	12
35		Sigma charge	12
36		Pi charge	12
37		Total charge	12
38		Sigma electronegativity	12
39		Pi electronegativity	12
40		Lone pair electronegativity	12
41		Polarizability	12
42		VC/2003 partial atom charges	12
43	Radial Distribution Function	Atom identity	48
44		Sigma charge	48
45		Pi charge	48
46		Total Charge	48
47		Sigma electronegativity	48
48		Pi electronegativity	48
49		Lone pair electronegativity	48
50		Polarizability	48
51		VC/2003 partial atom charges	48
52	Radial Distribution Function weighted with van der Waals surface area	Atom identity	48
53		Sigma charge	48
54		Pi charge	48
55		Total charge	48
56		Sigma electronegativity	48
57		Pi electronegativity	48
58		Lone pair electronegativity	48
59		Polarizability	48
60		VC/2003 partial atom charges	48
		Total	1284

S2. Analyzing the Overlap of Optimal Descriptor Sets Reveals Extent of Similarity

Descriptor selection identified optimized descriptor sets for each PubChem data set and machine learning algorithm pairing. The following experiment analyses the pair-wise overlap of optimized descriptor sets for each PubChem dataset using Tanimoto coefficients [4] (Figure S1). The average overlap ranges from 0.01 to 0.31 when comparing different ML algorithms on one PubChem dataset. This is indicative of chosen descriptor sets having only few descriptors in common for a pair of machine learning algorithms. Individual off-diagonal values never exceed a Tanimoto coefficient of 0.47 suggesting that two optimized descriptor sets have generally less than half of their descriptor values in common for a particular SAID. Also, the size of the optimal descriptor set varies for every ML algorithm. The diagonal values in Figure S1 display the recovered descriptors in comparison to the entire available 1,284 descriptor values. Naïvely, one might expect a strong dependence on the target data set. Thus, the relation between chemical structure and biological activity can be established using multiple different combinations of descriptors—a finding that can be explained by inter-dependence among different descriptor sets.

Figure S1. Pairwise comparison of optimized descriptor sets among machine learning algorithms trained on the same PubChem data set. Each dataset (SAID) is represented with a heat map reporting the Tanimoto coefficients of element overlap for two ML methods shown in rainbow coloring. 0.0 (green) represents no overlap while 1.0 (red) indicates full overlap. The average percent overlap is depicted in blue taking off-diagonal values into account. All optimal descriptor sets determined by SFFS. The diagonal values (white) are excluded from the average calculation and represent the percent overlap of the optimized descriptor set compared to the initial set containing 1,284 descriptors.

SAID 435008

ANN	DT	KN	SVM		
22%	0.25	0.17	0.18	0.20	ANN
0.25	33%	0.30	0.23	0.26	DT
0.17	0.30	16%	0.16	0.21	KN
0.18	0.23	0.16	73%	0.19	SVM
0.20	0.26	0.21	0.19		

SAID 1798

ANN	DT	KN	SVM		
16%	0.01	0.01	0.14	0.05	ANN
0.01	14%	0.29	0.30	0.20	DT
0.01	0.29	7%	0.15	0.15	KN
0.14	0.30	0.15	22%	0.20	SVM
0.05	0.20	0.15	0.20		

SAID 435034

ANN	DT	KN	SVM		
26%	0.26	0.06	0.20	0.17	ANN
0.26	42%	0.24	0.20	0.23	DT
0.06	0.24	38%	0.33	0.21	KN
0.20	0.20	0.33	40%	0.25	SVM
0.17	0.23	0.21	0.25		

SAID 2258

ANN	DT	KN	SVM		
26%	0.11	0.01	0.36	0.16	ANN
0.11	10%	0.06	0.26	0.14	DT
0.01	0.06	6%	0.12	0.06	KN
0.36	0.26	0.12	30%	0.24	SVM
0.16	0.14	0.06	0.24		

SAID 1843

ANN	DT	KN	SVM		
26%	0.06	0.27	0.40	0.24	ANN
0.06	45%	0.04	0.06	0.05	DT
0.27	0.04	9%	0.15	0.15	KN
0.40	0.06	0.15	20%	0.20	SVM
0.24	0.05	0.15	0.20		

SAID 463078

ANN	DT	KN	SVM		
19%	0.18	0.01	0.27	0.15	ANN
0.18	17%	0.02	0.18	0.12	DT
0.01	0.02	0.3%	0.01	0.01	KN
0.27	0.18	0.01	69%	0.15	SVM
0.15	0.12	0.01	0.15		

SAID 488997

ANN	DT	KN	SVM		
21%	0.26	0.17	0.10	0.18	ANN
0.26	16%	0.14	0.06	0.15	DT
0.17	0.14	83%	0.27	0.19	KN
0.10	0.06	0.27	32%	0.14	SVM
0.18	0.15	0.19	0.14		

SAID 2689

ANN	DT	KN	SVM		
49%	0.23	0.25	0.25	0.24	ANN
0.23	17%	0.21	0.02	0.15	DT
0.25	0.21	38%	0.26	0.24	KN
0.25	0.02	0.26	34%	0.18	SVM
0.24	0.15	0.24	0.18		

SAID 485290

ANN	DT	KN	SVM		
52%	0.23	0.47	0.23	0.31	ANN
0.23	27%	0.16	0.16	0.18	DT
0.47	0.16	59%	0.29	0.30	KN
0.23	0.16	0.29	32%	0.23	SVM
0.31	0.18	0.30	0.23		

Figure S2 compares descriptor set overlap from the perspective of each machine learning algorithm in the same fashion as in Figure S1. Again, only few descriptors overlap. The extend of percent overlap compared to the initial 1,284 descriptor values ranges from 0.3% to 83% (KN), 16% to 52% (ANN), 10% to 45% (DT), and 20% to 72% (SVM). ANNs and DTs tend to choose a more compact descriptor set then KNs. SVMs show a slightly increased descriptor set in comparison. The results suggest that the optimal descriptor subset is strongly dependent on both machine learning method and individual data sets.

Figure S2. Heat maps reporting Tanimoto coefficients of descriptor overlap on SFFS optimized descriptor sets among the various machine learning algorithms trained on different PubChem data sets (SAID). 0.0 (green) indicates no overlap while 1.0 (red) indicates full overlap. The average overlap is depicted in blue. All optimal descriptor sets were determined by SFFS. The diagonal values (white) are excluded from the average calculation and represent the percent overlap of the optimized descriptor set compared to the initial set containing 1,284 descriptors.

ANN

SAID	1798	1843	2258	2689	435008	435034	463087	485290	488997	
1798	16%	0.10	0.10	0.13	0.28	0.15	0.01	0.11	0.18	0.13
1843	0.10	26%	0.10	0.21	0.08	0.22	0.22	0.20	0.22	0.17
2258	0.10	0.10	26%	0.02	0.30	0.10	0.11	0.02	0.13	0.11
2689	0.13	0.21	0.02	49%	0.11	0.21	0.13	0.65	0.07	0.19
435008	0.28	0.08	0.30	0.11	22%	0.13	0.15	0.11	0.21	0.17
435034	0.15	0.22	0.10	0.21	0.13	26%	0.26	0.26	0.19	0.19
463087	0.01	0.22	0.11	0.13	0.15	0.26	19%	0.18	0.13	0.24
485290	0.11	0.20	0.02	0.65	0.11	0.26	0.18	52%	0.15	0.21
488997	0.18	0.22	0.13	0.07	0.21	0.19	0.13	0.15	20%	0.16
	0.13	0.17	0.11	0.19	0.17	0.19	0.15	0.21	0.16	

DT

SAID	1798	1843	2258	2689	435008	435034	463087	485290	488997	
1798	14%	0.16	0.00	0.19	0.19	0.11	0.04	0.10	0.01	0.10
1843	0.16	45%	0.23	0.12	0.39	0.48	0.24	0.31	0.16	0.26
2258	0.01	0.23	10%	0.01	0.15	0.06	0.20	0.05	0.04	0.09
2689	0.19	0.12	0.01	17%	0.29	0.18	0.15	0.30	0.03	0.16
435008	0.19	0.39	0.15	0.29	33%	0.41	0.37	0.27	0.16	0.28
435034	0.11	0.48	0.06	0.18	0.41	42%	0.30	0.36	0.17	0.26
463087	0.04	0.24	0.20	0.15	0.37	0.30	18%	0.17	0.13	0.29
485290	0.10	0.31	0.05	0.30	0.27	0.36	0.17	27%	0.28	0.23
488997	0.01	0.16	0.04	0.03	0.16	0.17	0.13	0.28	16%	0.12
	0.10	0.26	0.09	0.16	0.28	0.26	0.20	0.23	0.12	

KN

SAID	1798	1843	2258	2689	435008	435034	463087	485290	488997	
1798	6%	0.01	0.44	0.04	0.05	0.01	0.01	0.08	0.08	0.09
1843	0.01	9%	0.01	0.19	0.01	0.19	0.01	0.14	0.11	0.08
2258	0.44	0.01	6%	0.02	0.01	0.02	0.03	0.08	0.07	0.08
2689	0.04	0.19	0.02	38%	0.10	0.44	0.01	0.33	0.36	0.19
435008	0.05	0.01	0.01	0.10	16%	0.12	0.01	0.16	0.12	0.07
435034	0.01	0.19	0.02	0.44	0.12	38%	0.01	0.42	0.40	0.20
463087	0.01	0.01	0.03	0.01	0.01	0.01	0.3%	0.01	0.01	0.12
485290	0.08	0.14	0.08	0.33	0.16	0.42	0.01	59%	0.62	0.23
488997	0.08	0.11	0.07	0.36	0.12	0.40	0.01	0.62	83%	0.22
	0.09	0.08	0.08	0.19	0.07	0.20	0.01	0.23	0.22	

SVM

SAID	1798	1843	2258	2689	435008	435034	463087	485290	488997	
1798	23%	0.21	0.19	0.07	0.21	0.29	0.21	0.07	0.26	0.19
1843	0.21	20%	0.18	0.29	0.24	0.18	0.25	0.34	0.17	0.23
2258	0.19	0.18	30%	0.17	0.39	0.25	0.39	0.26	0.34	0.27
2689	0.07	0.29	0.17	35%	0.37	0.19	0.32	0.15	0.34	0.24
435008	0.21	0.24	0.39	0.37	72%	0.28	0.68	0.26	0.26	0.34
435034	0.29	0.18	0.25	0.19	0.28	40%	0.36	0.15	0.26	0.25
463087	0.21	0.25	0.39	0.32	0.68	0.36	69%	0.27	0.27	0.34
485290	0.07	0.34	0.26	0.15	0.26	0.15	0.27	32%	0.13	0.20
488997	0.26	0.17	0.34	0.34	0.26	0.26	0.27	0.13	32%	0.25
	0.19	0.23	0.27	0.24	0.34	0.25	0.34	0.20	0.25	

S3. Complete Representation of Inactive Compounds Improves QSAR Model Precision for Initial True-Positive Range

Generally, experimental HTS datasets are highly unbalanced, *i.e.*, the number of active compounds is much smaller than the number of inactive compounds. This poses a challenge for training predictors with naïve objective functions as—for example—99% of all compounds are classified correctly by calling all compounds "inactive" and the root mean square difference (RMSD) between experimental and predicted activity will be low. To circumvent this problem all training data sets were balanced—*i.e.*, an equal number of active and inactive molecules were used. This can be achieved through over-sampling of active or under-sampling of inactive molecules. The first strategy utilizes all data for training while the second strategy only uses part of the inactive molecules, a strategy that could be attractive in order to accelerate the training procedure through substantially smaller datasets in the absence of GPU-acceleration. Note that the number of inactive compounds in the independent data set was not modified, i.e. the QSAR model still needs to classify all compounds of the independent dataset correctly. The percentage of inactive compounds reserved for training and monitoring data set partitions was systematically increased ranging from 1%, 5%, 10%, 20%, 30% … 100% for three selected datasets with SAIDs 488997, 485290, and 2258. Each data set contains more than 300,000 inactive compounds total. Adding more inactive compounds to the training and monitoring dataset increased the integral of the TNR-TPR curve for the initial TPR range compared to the 1% case (see Figure S3). A higher TNR-TPR curve indicates a high TP to FP ratio (FP=1-TN), implies a high precision and therefore a high Enrichment. Evaluating the TPR range of 0.0 to 0.2, QSAR models with more inactives included in the training and monitoring data showed a higher precision for identifying active compounds compared to the 1% case. These results suggest a similar overall performance for all training data configurations but higher accuracy for the initial TPR range if more inactives are involved.

Figure S3. The TNR-TPR curve is shown for SAIDs 488997, 485290, and 2258. Each curve represents a QSAR model trained on 1%, 5%, 10% … 100% of the given monitoring and training data. The plots on the right-hand side show an enlarged view of the initial portion of the true-positive rate. With increasing percent of the used monitoring and training data the color intensity of each curve decreases. (1% - dark, 100% - light).

SAID 488997

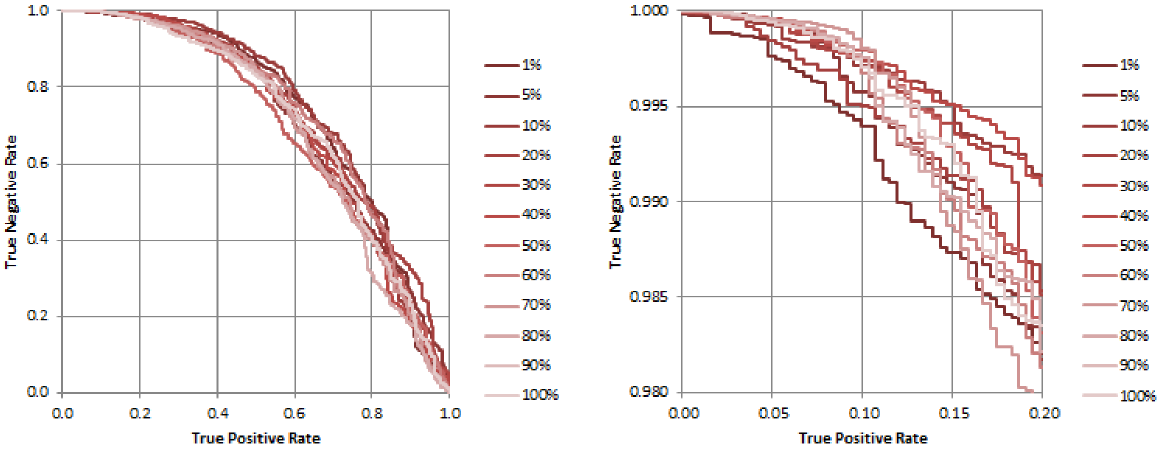

Figure S3. *Cont.*

SAID 485290

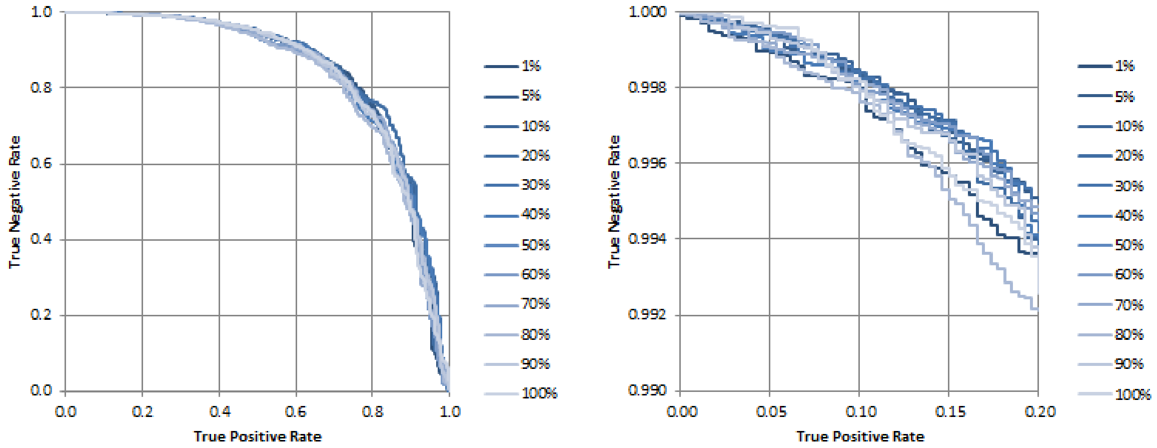

SAID 2258

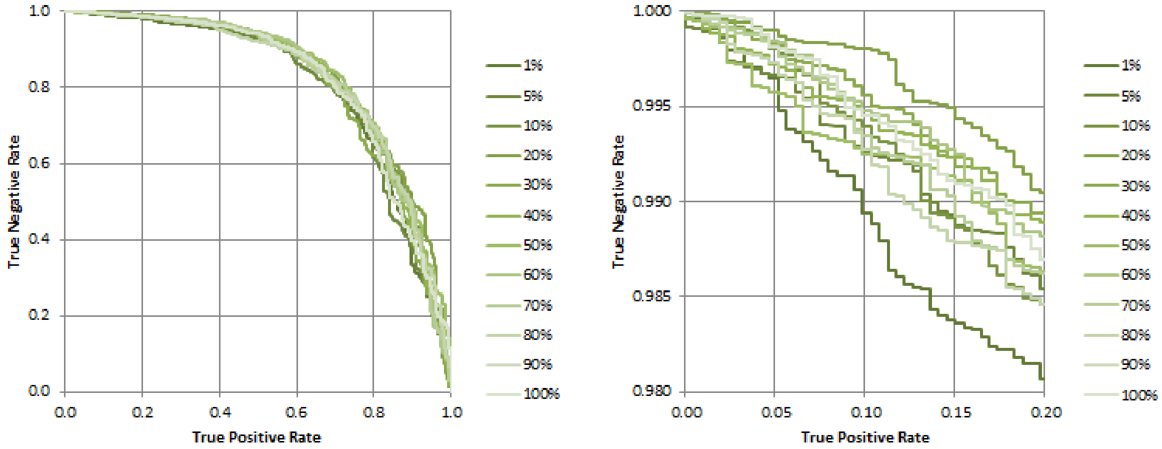

S4. Protocol Capture

The QSAR modeling framework BCL::ChemInfo and its applications can be licensed free for academic use through www.meilerlab.org/bclcommons. We provide sample command lines to capture the execution protocol tested on a Linux CentOS 5 operating system available at www.meilerlab.org/qsar_pubchem_benchmark_2012.

References

1. Gilson, M.K.; Gilson, H.S.R.; Potter, M.J. Fast Assignment of Accurate Partial Atomic Charges: An Electronegativity Equalization Method that Accounts for Alternate Resonance Forms. *J. Chem. Inform. Comput. Sci.* **2003**, *43*, 1982–1997.
2. Caballero, J.; Fernandez, M.; Gonzalez-Nilo, F.D. Structural requirements of pyrido[2,3-d]pyrimidin-7-one as CDK4/D inhibitors: 2D autocorrelation, CoMFA and CoMSIA analyses. *Bioorg. Med. Chem.* **2008**, *16*, 6103–6115.
3. Gonzalez, M.P.; Teran, C.; Teijeira, M.; Helguera, A.M. Radial distribution function descriptors: An alternative for predicting A2 A adenosine receptors agonists. *Eur. J. Med. Chem.* **2006**, *41*, 56–62.
4. Lipkus, A. A proof of the triangle inequality for the Tanimoto distance. *J. Math. Chem.* **1999**, *26*, 263–265.
